# Pellets enriched with healthy hay and quebracho are not sufficient to control gastrointestinal nematodes in meat sheep commercial flocks

**DOI:** 10.1017/S0031182024001409

**Published:** 2024-10

**Authors:** Léa Bordes, Corentin Souchon, Alice Claessens, Sophie Lavigne, Geneviève Bouix, Margaux Goyenetche, Laurence Sagot, Christelle Grisez, Guy-Gérard Merlande, Philippe Jacquiet

**Affiliations:** 1 IHAP, UMR 1225 INRAE/ENVT, Université de Toulouse, Toulouse, France; 2 GEODE, Coopérative ovine, Roussines, France; 3 Centre Interrégional d'Information et de Recherche en Production Ovine (CIIRPO), Saint-Priest-Ligoure, France

**Keywords:** condensed tannins, gastro-intestinal nematodes, sheep

## Abstract

The emergence of AH multiresistant GIN compromises sustainability of grassland sheep farming worldwide. Plants rich in condensed tannins are an alternative method of parasitism management that is currently being explored. Feed supplementation trials with pellets rich in sainfoin (*Onobrychis viciifolia*) and quebracho (*Schinopsis* spp.) were carried out. Three meat sheep farms in western France took part in the study and a total of 4 trials were carried out.

During these 21-day trials, the ewes were returned to the sheepfold and half of them received a balanced ration supplemented with 70 g day^−1^ of healthy hay and quebracho pellets, while the other half received the same ration supplemented with 70 g day^−1^ of lucerne pellets. Fecal egg counts (FEC) were carried out at the start and end of each trial, and nematode species were identified by real-time PCR after larval culture. At D0, FEC were similar in both groups for all 4 trials. Proportions of species infecting the ewes varied from 1 trial to another: *Haemonchus contortus* was predominant in summer and *Trichostrongylus colubriformis* in winter. At D21, there were no significant differences in FEC between groups. Helminthofauna were not significantly different between groups, except for 1 trial where the proportion of *H. contortus* was reduced in the group supplemented with condensed-tannin pellets. The use of condensed tannins still requires additional studies to be advised as an effective method to manage gastrointestinal nematodes in farm.

## Graphical abstract

**Figure:**
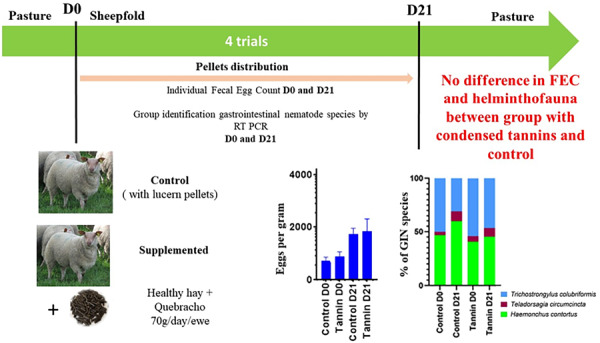

## Introduction

In southwestern France, the use of pasture for most of the year is the rule in dairy and meat sheep systems. This has many advantages in terms of food availability, costs saving for farmers and animal welfare. However, the main concern with natural grazing is the gastrointestinal nematodes (GIN) infection of animals by. These parasites lead to losses in milk, meat and wool production, sometimes mortality and also impact adult reproductive performances (Mavrot *et al*., 2015).

In France, 3 GIN species are considered to be dominant according to their pathogenesis and relative abundances: *Haemonchus contortus*, *Teladorsagia circumcincta* and *Trichostrongylus colubriformis*. In order to control these GIN species, farmers usually have recourse to chemical anthelminthic treatments which are easy-to-use and inexpensive and are supposed to be effective. However, repeated and systematic treatments during decades have led to the emergence of anthelmintic resistance (Falzon *et al*., 2014). The first family of anthelmintic molecules which was impacted by resistance was the benzimidazoles (BDZ). Widely used since the 1960s, the resistance of GIN to this family is now widely spread in Europe (Vernerova *et al*., 2009; Papadopoulos *et al*., 2012) and in France (Chartier *et al*., 1998; Geurden *et al*., 2014; Krücken *et al*., 2024). To overcome this situation, a switch to macrocyclic lactones (ML) family had been made in both dairy and meat sheep farms. Nevertheless, this change of family was not accompanied by a change in treatment's practices and resistance to ML appeared very quickly (Borgsteede *et al*., 2007; Papadopoulos, 2008; Scheuerle *et al*., 2009). Thus, recently, the first cases of multiresistance of GIN to several families of anthelmintics were reported in Europe (Sargison *et al*., 2007; Traversa *et al*., 2007; Taylor *et al*., 2009; Voigt *et al*., 2012; Van den Brom *et al*., 2013; Krücken *et al*., 2024) including France (Paraud *et al*., 2016; Milhes *et al*., 2017; Cazajous *et al*., 2018; Bordes *et al*., 2020; Jouffroy *et al*., 2023).

To face the spread of anthelminthic resistance in GIN, alternative or complementary solutions have been studied and are based on 3 main actions: targeting the parasites in the host (targeting selective treatment, bioactive plants…) (Ahuir-Baraja *et al*., 2021), increasing the host's resistance (protein supplementation, genetic selection of host resistance…) (Aguerre *et al*., 2018; Thorne *et al*., 2024) and improving the pasture's management (cell grazing, pasture rotation…) (Ruiz-Huidobro *et al*., 2019). One of the alternatives to chemical anthelmintic treatments is the use of bioactive plants, showing anthelminthic effects. This is a short-term solution as bioactive plants are acting directly on the parasites. Their anthelminthic effect is mainly based on their ability to produce tannins, which are secondary metabolites, divided in 2 main groups : hydrolysable tannins and condensed tannins (CTs), the latter only being involved in the control of GIN (Hoste *et al*., 2006). CTs are widespread in the plant kingdom as they are found in both angiosperms and gymnosperms (Stafford and Lester, 1980). The highest levels of CTs are found in legumes such as healthy hay (*Onobrychis viciifolia*), bunched hedizarum (*Sulla coronaria*), birdsfoot trefoil and big trefoil (*Lotus corniculatus L.* and *Lotus pedunculatus*) and Sericea lespedeza (*Lespedeza cuneata*) (Mueller-Harvey *et al*., 2019). Other forbs such as chicory (*Cichorium intybus*) and plantain (*Plantago lanceolata*) may also have interesting tannin contents. Finally, some chestnut barks, such as quebracho (*Schinopsis* spp.), have high CTs content (Athanasiadou *et al*., 2001), which can supplement or increase tannin intake in animals using barks extracts in diet.

Several studies showed the effect of CTs in GIN in *in vitro* conditions: inhibition of the exsheathment of L3 larvae (Brunet *et al*., 2007) associated with disruptions between cuticle and sheath (Brunet *et al*., 2011) and decreased larval motility (Paolini *et al*., 2004; Manolaraki *et al*., 2010). In *vivo* studies on sheep and goats have also been carried out to understand the mechanisms of action of tannins: exposure of worms to tannins result in a decrease of motility in adult worms (Paolini *et al*., 2004), a reduction of the number of eggs in the uterus of adult female worms (Paolini *et al*., 2003a; Heckendorn *et al*., 2006, 2007; Manolaraki *et al*., 2010; Gaudin *et al*., 2016) and a decrease of fecal egg excretion (Paolini *et al*., 2003a, 2003b; Manolaraki *et al*., 2010). These effects were observed on susceptible as well as multiresistant GIN populations (Gaudin *et al*., 2016). When CTs and infective larvae were given simultaneously in experimental studies, the number of adult worms established in the digestive system was decreased for *T. colubriformis and H. contortus* compared to control groups (Paolini *et al*., 2003b; Heckendorn *et al*., 2006). Initially, CTs extracts or hay were used in these previous studies. In order to improve the conservation and stability of CTs and facilitate the distribution to animals and the storage in farms, pellets were developed with healthy hay (Terrill *et al*., 2007; Gaudin *et al*., 2016) and *Sericea lespedeza* (Gujja *et al*., 2013; Kommuru *et al*., 2014, 2015) with expected anthelminthic effects.

According to these encouraging experimental results, it is now essential to test CTs effect on field conditions with a simple protocol for sheep breeders. Three commercial farms from the western France, a region undergoing strong GIN pressure and multi-resistant nematodes were selected in this study. We evaluated the impact of a diet complementation based on commercial pellets enriched with healthy hay and quebracho on the intensity of GIN egg excretions and on the composition of GIN communities in animals naturally infected.

## Materials and methods

### Experimental design

In order to propose practical solutions to sheep breeders, we chose commercially available tannin-rich plant pellets (Natuviamix©, Mg2Mix), to be tested in this study. These pellets have the interest to be more concentrated in CTs than their counterparts composed of healthy hay only, thanks to the presence of quebracho extracts in the pellets with 30% of CTs.

A total of 4 trails were performed on ewes from 3 different farms during the year 2021. Farm 1 and Farm 2 were two commercial sheep farms of the *Rouge de l'Ouest* (RdO) meat sheep breed and Farm 3 was a flock of the *Brebis Vendéenne* (V) meat sheep breed. Farm 3 belongs to a research center for sheep breeding, the ‘Centre InterRegional d'Information et de Recherche en Production Ovine’ (CIIRPO). All farms are located in Western France, in *Deux-Sèvres, Vienne* and *Haute-Vienne French ‘départements’*. In farms 1 and 2, the ewes used for the study were 2.5 years old and from 2 to 5 years old on the farm 3. In farm 1, 50 ewes were recruit for trials, 51 in farms 2 and 58 in farms 3. Ewes were allowed to graze during 7 to 8 months per year and were maintained indoors twice a year: once in the summer time (2 months) when the grass quantity was no longer sufficient to feed them and once in the winter (2 to 3 months), during lambing period. In the sheepfold, they had a balanced diet covering their nutritional needs. For trial A, B and D the ewes were neither in gestation nor lactating. In trial C, the ewes were at the end of gestation and the beginning to the lambing period.

The trials were performed at the beginning of these indoors periods, on naturally infected ewes. The ewes did not receive any anthelminthic treatment at least 2 months preceding the trails. The numbers of ewes and their description for each trial are presented in Table 1. In each trial, ewes were separated into 2 groups: 1 group received the tannin-rich pellets (Tannin Group) and the other group received lucerne pellets (Control Group). Pellets were distributed daily during 21 days, on a base of 70 g day ewe^−1^. The daily amount of distributed pellets corresponded to the manufacture's recommendations but the duration of distribution (21 days) was longer than those recommended (10 days only), according with previous successful studies in small ruminants (Paolini *et al.*, 2005; Heckendorn *et al.*, 2006). The diets were equivalent in both groups, and provided the same nutritional intake (protein and energy) to the ewes.

**Table 1.** Description of the experimental design

Table 1	Farm 1		Farm 2				Farm 3	
	Trial A		Trial B		Trial C		Trial D	
Period of cure	Summer 2021		Winter 2021		Summer 2021		Summer 2021	
Age of ewes during cure (years)	2.5		2		2.5		From 2 to 6 (mean = 3)	
Total number of ewes	50		51		37		58	
Group	Control	Tannin	Control	Tannin	Control	Tannin	Control	Tannin
Number of ewes per group	22	28	23	28	19	18	29	29

Control group, ewes receiving lucerne supplementation; Tannin group, ewes receiving tannin-rich pellets supplementation.

At the beginning of each trial (D0), the ewes received their first ration with pellets and individual fecal samples were collected.

### Fecal egg counts and molecular identification of GIN species

Individual fecal egg counts (FEC) were performed by a modified McMaster technique, using a saturated sodium chloride solution of density 1.2 (Raynaud *et al*., 1970). This technique has a sensitivity of 15 eggs per gram (EPG). The remaining feces were pooled by trial and by group, to obtain L3 larvae in composite larval cultures. All composite larval cultures were incubated in a beaker for 12 days at 24 ± 1°C and humidified every 2 days with tap water. Third stage larvae (L3s) were recovered by filling the beaker with tap water at room temperature (±25°C) and inverting it on a Petri dish (MAAF, 1986) filled with tap water. L3 larvaes have migrated into the water contained in the petri dish and were collected every 12 h, in 3 passes in a same volume of 40–45 mL. Overnight decanting at 4°C in fridge concentrated L3 larvaes at the bottom of the tube. The supernatant was discarded, leaving only a 5 mL volume of larval suspension. These suspensions were stored at 4°C until identification step.

Identifications of larvae were done by real-time PCR at the beginning and at the end of each 21 days' trial. Briefly, composite larval cultures were incubated for 12 days at 24 ± 1°C in jars and humidified every 2 days with tap water. After incubation, the jars were filled with tap water and inverted onto a square Petri dish, also filled with tap water and left for 2 days at room temperature (±24°C). The L3 larvae were recovered every 12 h by collecting the water in the Petri dish. They were concentrated in a 40 to 50 mL Falcon tube and placed in a refrigerator (±4°C) overnight to concentrate the larvae at the bottom of the tube. The supernatant was removed to obtain a larval suspension in a final volume of 5 mL, stored at 4°C until analysis. GIN species molecular identification was performed by real-time PCR (Milhes *et al*., 2017). This technique specifically detects and quantifies larvae of *H. contortus*, *T. circumcincta* and *T. colubriformis*.

### Data and statistical analysis

The distribution of the data and their descriptive analysis were obtained using R software, version 4.2.1 (2022-06-23) (R core Team, 2023) and R Studio software version 2022-07-01 (Posit Team, 2022). Graphs were made with GraphPad Prism version 8.0.1 for Windows (GraphPad Software, 2023).

Different variables were analysed on data collected at D0 and D21:

- EPG at D0 and D21, as a continuous quantitative variable, showed an asymmetrical distribution
- Supplementation, qualitative variable has been divided into 2 categories: ‘Control’ for the group with lucerne supplementation and ‘Tannin’ for the group with the tannin-rich pellets supplementation

Several levels of analysis were carried out to assess the anthelmintic effect of pellets rich in CTs on FEC at D0 and D21. Firstly, an overall analysis was carried out, bringing together the results from all the trials. Each condition tested had a number greater than 30 individuals. After an evaluation of the homoscedasticity of the variances using a Fligner test, a Student test was carried out in the case of equal variances and a Welch test in the case of unequal variances. Secondly, statistical analyses for each trial were carried out using non-parametric tests (Wilcoxon test, Kruskall-Wallis test).

The GIN species were divided into 3 categories according to the species identified in RT-PCR: ‘*Haemonchus contortus*’, ‘*Teladorsagia circumcincta*’, ‘*Trichostrongylus colubriformis*’. The proportions of each species were compared at D0 and D21 by *χ*^2^ test for each trial and for the global analysis, composed with the mean of proportion of each species. *χ*^2^ tests were corrected when necessary with Yates correction (at least one theoretical number less than 5).

## Results

**Figure 1.**
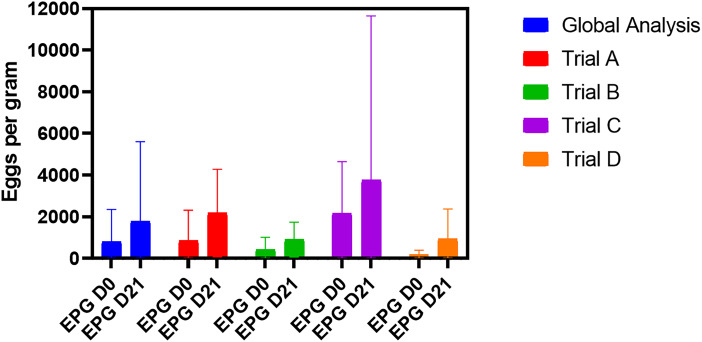
Intensities of egg excretions (means and standard deviations; pooled data from control and tannins groups) in the different trials and for the whole study.

The mean FEC (tannins and control groups pooled) at D0 and D21 for the global experiment or for each individual trial are presented in Fig. 1. Intensities of egg excretions at the beginning of the different trials were variable: in trial D, the mean FEC was relatively low (166 EPG), while in trial C, high excretions were noticed (2167 EPG). At D21, we noticed an increase of mean FEC in each trial.

**Figure 2.**
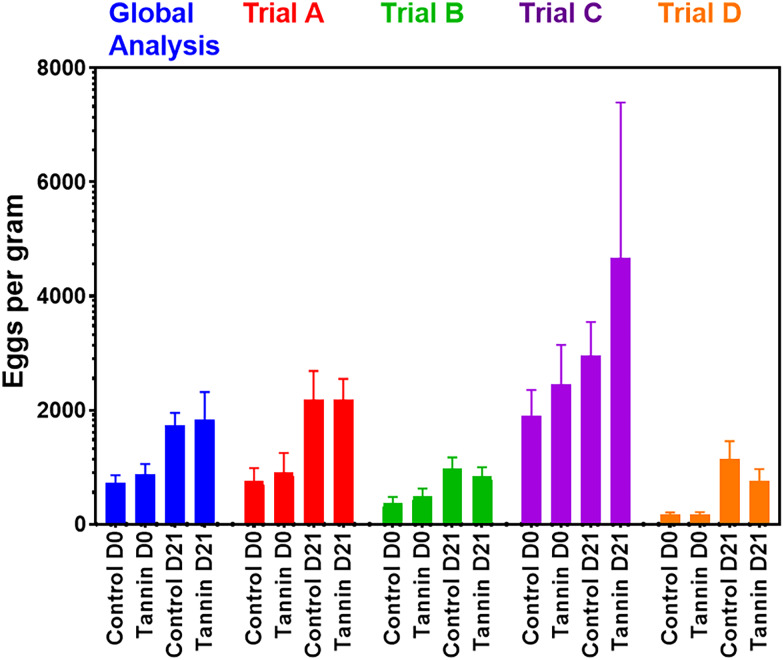
Intensities of egg excretions (Means and standard deviations) per group in the different trials and in pooled data.

The effect of supplementation was evaluated by comparing the FEC at D0 (beginning of the indoors period and the cure) and D21 (end of the cure) between the 2 groups (control *vs* tannin) of ewes (Fig. 2). At D0, there was no significant difference in the intensity of GIN egg excretions between the 2 groups of supplementation, whatever the trial and for the global analysis [*P* = 0.55, confidence interval (CI) (−600 to 302)). Moreover, no significant difference between FEC of the 2 groups was reported at the end of the trial (all *P* values > 0.3), whatever the trial including the global analysis (*P* = 0.85, CI [−1151 to 955]).

**Figure 3.**
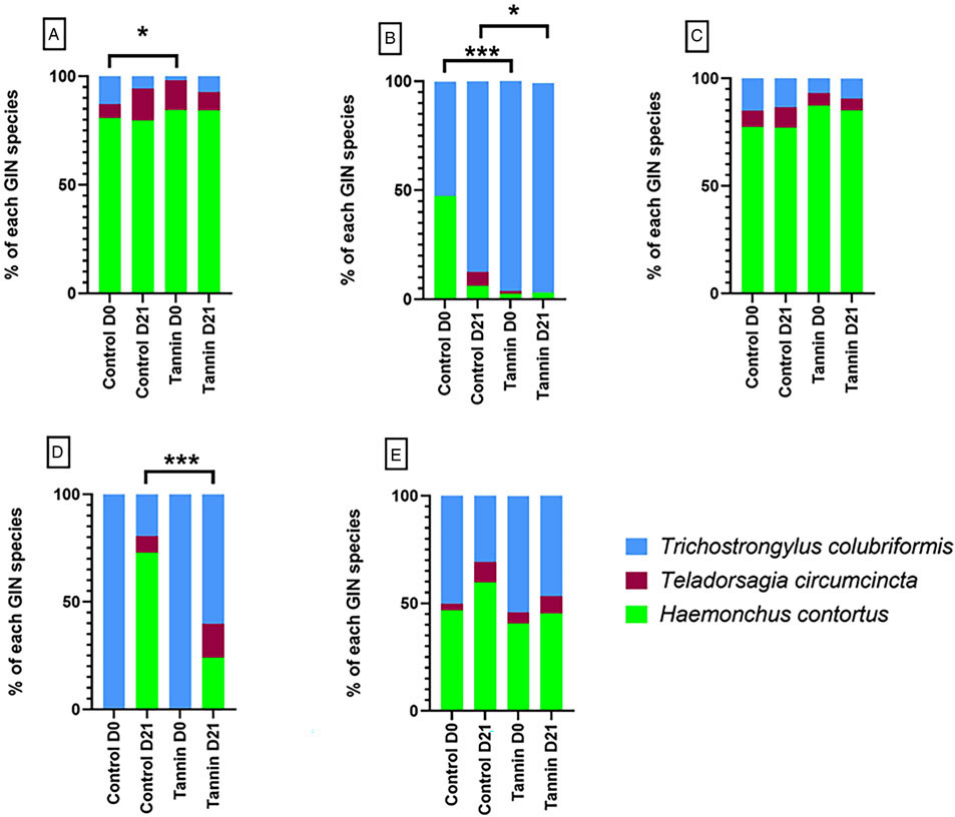
Relative abundances of the three gastrointestinal nematodes species (*Haemonchus contortus*, *Teladorsagia circumcincta* and *Trichostrongylus colubriformis*) determined by real-time PCR: (A) helminth community for trial A (summer), (B) helminth community for trial B (winter), (C) helminth community for trial C (summer), (D) helminth community for trial D (summer) and (E) global helminth community, with all trials are pooled. (* *P* < 0.05, ** *P* < 0.01, *** *P* < 0.001).

The proportions of GIN species (Fig. 3) in each treatment group and trial were investigated. Helminth communities revealed the presence of the 3 GIN species in all trials but in different proportions. Grossly, *H. contortus* was the dominant species in summer in farms 1 and 2 (trial A, Fig. 3B and trial C, Fig. 3D), excepted in farm 3. By contrast, *T. colubriformis* was the main species in farm 2 during winter. The results obtained in farm 3 (summer time) are interesting because *T. colubriformis* is the only species found at D0 but the relative importance of *H. contortus* is increasing from D0 to D21 (Fig. 3E). *χ*^2^ tests performed to compare relative abundances of GIN species showed variable results according to the trial. At D0, helminth communities were significantly different between the control and tannin groups on the trial A (*χ*^2^ = 11.2; 2 d.f; *P* = 0.03) and trial B (*χ*^2^ = 54.18; 2 df; *P* = 1.7 × 10^−12^). At D21, only trial B (*χ*^2^ = 6.53; 2 df; *P* = 0.038) and trial D (*χ*^2^ = 48.2, 2 df; *P* = 3.4 × 10^−11^) showed significant differences in helminth communities, characterized by an increased proportion of *T. colubriformis* and/or *T. circumcincta* in tannin group.

## Discussion

This study evaluated the impact of a CTs-rich diet on FEC and as a possible alternative mode of control of GIN in commercial meat sheep farms. Commercially available pellets claiming anthelminthic activity were used at the recommended daily amount but for a double duration (21 days instead of 10 days). It is one of the few studies conducted on commercial flocks naturally infected with GIN and evaluating the effects of CTs supplementation on intensities of egg excretions and on the composition of helminth communities.

In this study, the use of pellets rich in condensed tannins was chosen over hay for several day-to-day management advantages. Pellets are easier to store and distribute to ewes, and their condensed tannin content is quality-assured by the manufacturer. In contrast, with healthy hay, this content can vary according to plant variety, number of harvest and drying (Kotze *et al*., 2009; Azuhnwi *et al*., 2011, 2013; Malisch *et al*., 2015), requiring farmers to measure the concentration of condensed tannins for each new harvest to adapt the distribution. In contrast, pellets are calibrated and dosed by the manufacturer, eliminating the need for recurrent analysis by the farmer.

The most striking result of this study was the absence of significant differences in FEC between ewes supplemented with tannins or lucerne in 4 trials. This result was obtained despite high intensities of GIN egg excretions (excepted for trial D at D0) and diversity in helminth communities. CTs have an effect on the installation and fecundity of adult GIN in host (Paolini *et al*., 2003b; Heckendorn *et al*., 2006; Manolaraki *et al*., 2010; Gujja *et al*., 2013; Gaudin *et al*., 2016). However, the direct evaluation of these parameters was not possible in our case, which leaded to the use of FEC as an indirect measure. Nonetheless, this measurement accurately represents the infection of animals by GIN (Cabaret *et al*., 1998) and the pastures contamination. Recently, impact of CTs on FEC have been studied also in horses and rabbits with pellets formulations. In both cases, CT-rich diet with pellets didn't show any effect on FEC (Legendre *et al*., 2017; Malsa *et al*., 2022). Interestingly, in Malsa and colleagues' study, healthy-hay pellets were from the same manufacturer and the same variety than in our study.

One of the major concern raised by the use of pellets is the influence of the technological transformation process (dehydration) on condensed tannins and the conservation of their properties. The majority of *in vivo* studies have focused on the use of tannin-rich plants as hay or directly as polyphenolic extracts (Paolini *et al*., 2004, 2005; Hoste *et al*., 2006; Manolaraki *et al*., 2010), particularly with healthy hay and/or quebracho, the component of the pellet tested in our study. Results of the activity of condensed tannins in the pellets seems linked with the temperature of the pelleting process : Legendre and colleagues (Legendre *et al*., 2017), showed reduced protein precipitation activity of CTs *in vitro* with a temperature of pelleting process around 80°C, while Terrill and colleagues (Terrill *et al*., 2007) do not exceed 70°C during the transformation process. In our case, the pellets tested came from the same manufacturer than in Legendre and colleagues study (Legendre *et al*., 2017), so there may also be a problem of reduced activity of CTs in this formulation. Moreover, the effect of temperature during processing has only been tested on CTs from healthy hay, so the effects on CTs contained in quebracho pellets are not known.

In addition to studying the impact of CTs on FEC, we were also interested in their impact on GIN communities and, in particular, on the proportions of the 3 main species found in French farms. In the present study, the main species were *H. contortus* and *T. colubriformis* which is rather expected in the region: the humid and warm weather is favouring the *H. contortus* populations during spring and early summer, while the cold winters favour *T. colubriformis* populations. On *in vivo* experimentation, CTs have showed an impact on each of the 3 means species (Paolini *et al.*, 2003b; Heckendorn *et al.*, 2006), but the effect in co-infection were poorly investigated: in the horse assay of Malsa and colleagues (Malsa *et al*., 2022), the nemabiome is not influenced by the CTs in the daily diet. In our case, helminth community were not systematically influence by CTs-rich diet, that suggest the effect is very weak.

The use of condensed tannins in the diet of small ruminants is of growing interest for the control of GIN, in a time of emergence of multiple anthelmintic resistance. While several studies have already showed a significant effect of condensed tannins on these parasites *in vitro* and in experimental infections, real-life trials with natural multi-species infections and carried out on commercial farms are much more unusual. In this study, we failed to demonstrate any effect of a pellet-based supplementation protocol for ewes on intensities of egg excretions nor in diversity of helminth communities. It is necessary to test new protocols on commercial farms in order to optimize the use of condensed tannins, particularly with regard to technical parameters such as cost, concentration of condensed tannins in diet, industrial process and the frequency and length of cure, before providing an effective and accessible control strategy to sheep breeders.

## Data Availability

Data available on request / reasonable request.
